# Supplementary material for: Repatterning of mammalian backbone regionalization in cetaceans
Source: Nat Commun. 2024 Aug 31;15:7587. doi: 10.1038/s41467-024-51963-w (PMC11365943; doi:10.1038/s41467-024-51963-w)
Supplement: Supplementary file 1 — Supplementary Information [file 41467_2024_51963_MOESM1_ESM.pdf]

Supplementary Information for:

Repatterning of mammalian backbone regionalization in cetacean

Amandine Gillet[1,2], Katrina E. Jones[1], Stephanie E. Pierce[2]

[1] *Department of Earth and Environmental Sciences, University of Manchester, Williamson Building, Oxford Road, Manchester M13 9PL, UK*

[2] *Museum of Comparative Zoology and Department of Organismic and Evolutionary Biology, Harvard University, 26 Oxford Street, Cambridge, MA 02138, USA*

<u>Contents :</u>

Supplementary Note: Sensitivity analysis of regionalization to vertebral count p.2

Supplementary Fig. 1. Nested regions hypothesis for cetacean backbone p.3

Supplementary Fig. 2. Vertebral measurements taken on each post-cervical vertebra p.4

Supplementary Fig. 3. Relationship between regionalization and disparity p.5

Supplementary Fig. 4. Variables contributions to PCO axes p.6

Supplementary Fig. 5. Relative position of anterior thoracic module boundary p.7

Supplementary Fig. 6. Average shape of vertebral centrum in modules p.8

Supplementary Fig. 7. Results of sensitivity analysis p.9

Supplementary Table 1. Results regressions on vertebral count, regionalization, and disparity p.10

Supplementary Table 2. Effect of habitat on region score and vertebral disparity p.10

Supplementary Table 3. Results regressions between regionalization and swimming speeds p.10

Supplementary Note: *Sensitivity analysis of regionalization to vertebral count*

Given the substantial variation in cetacean vertebral count, we investigated whether the optimal number of regions and associated region score were affected by the number of vertebrae. To this purpose, we simulated theoretical backbones with increasing predefined number of vertebrae and a predefined number of regions. Predefined number of vertebrae ranged from 35 to 60 with increments of 5 vertebrae and predefined number of regions ranged from 5 to 8. For each predefined number of vertebrae and regions, 25 different backbones were simulated, resulting in a total of 600 simulated backbones. For each simulated backbone, the region analysis was run on (1) all the vertebrae and (2) a subset of 33 equidistant vertebrae along the backbone.

For each analysis, the region score, obtained with BIC, was retained. Linear regressions were used to test the correlation between the number of vertebrae in the simulated backbones and region score for each predefined number of regions for analyses run on all vertebrae and on subsampled vertebrae separately.

Results are presented in Supplementary Fig. 7 and show that the recovered region score tends to increase significantly with the number of vertebrae when the analyses are run on all the vertebrae, indicating that more complex models will be selected with increasing vertebral count. However, the region score tends to remain stable (or to slightly decrease) with increasing vertebral count when analyses are performed on a constant number of subsampled vertebrae.

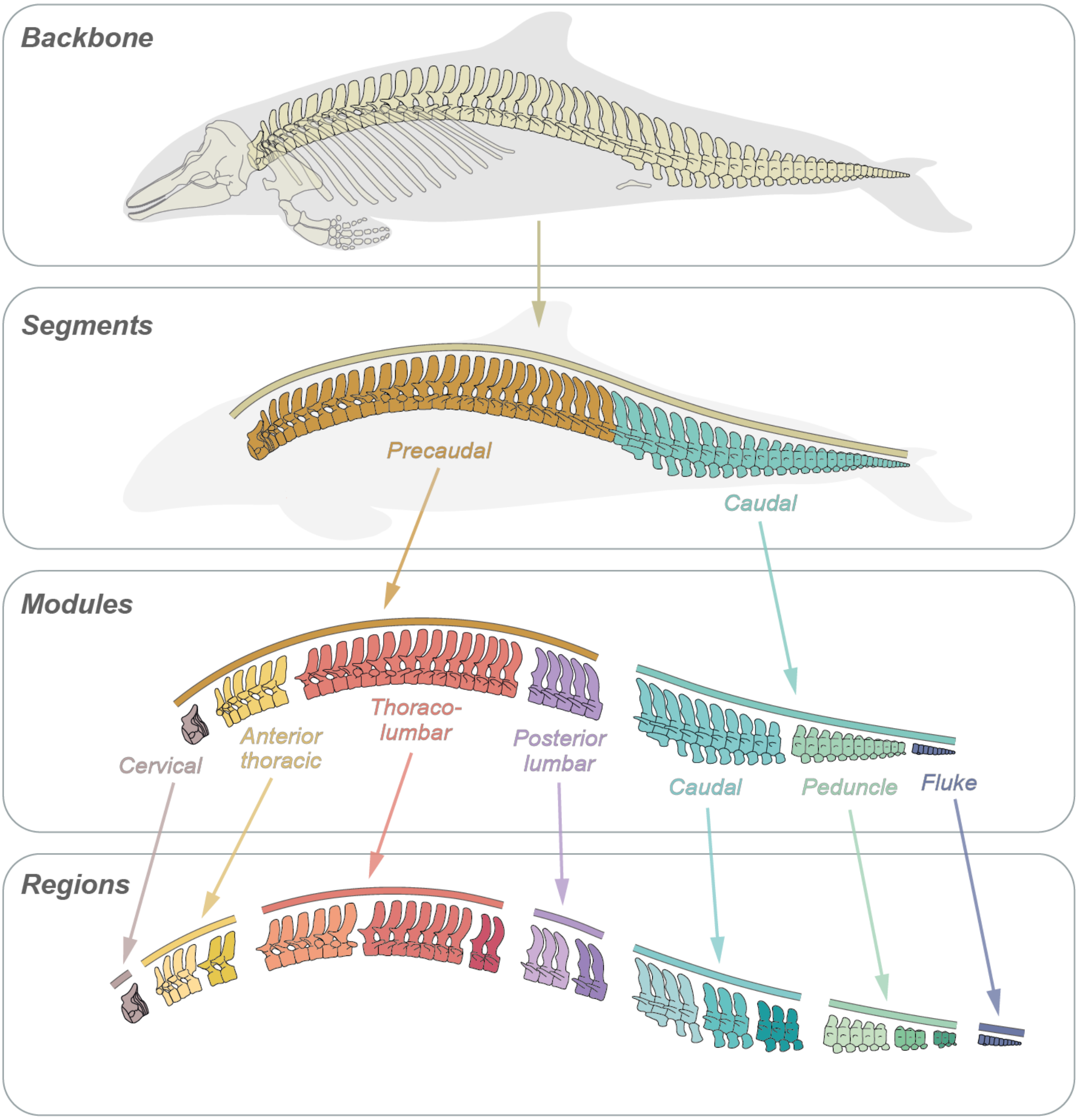

Supplementary Fig. 1. Nested regions hypothesis for cetacean backbone. Schematic illustration of the different organisational levels of the cetacean backbone. The backbone can be conveniently divided into precaudal and caudal segments, with the first caudal vertebra bearing a chevron on the caudo-ventral face of its vertebral centrum. Within each segment, we identify different modules: anterior-thoracic, thoraco-lumbar, and posterior lumbar in the precaudal segment, and caudal, peduncle, and fluke in the caudal segment. These modules are present in all cetaceans, with the exception of the posterior lumbar being identified only in some oceanic dolphins and porpoises. Finally, each module can be further subdivided into multiple regions, with a minimum of six and a maximum of nine post-cervical regions across all extant cetaceans. Note that the regions presented here correspond to the maximal number of regions present in each module and recovered in at least two species in our dataset and do not illustrate the regionalization of a given species.

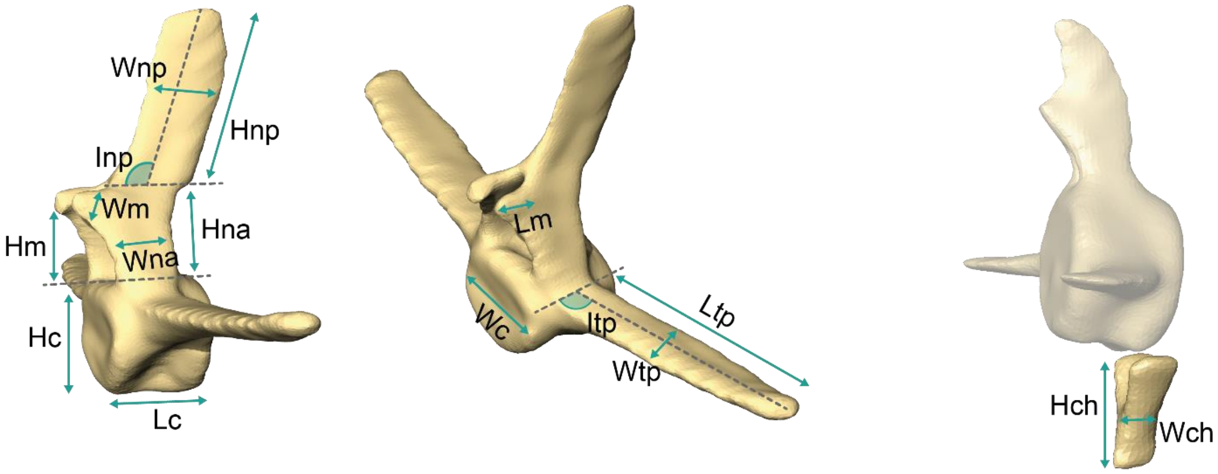

Supplementary Fig. 2. Vertebral measurements taken on each post-cervical vertebra. Fourteen linear and two angular measurements were taken on each vertebra. Acronyms key: The first letter of the acronym corresponds to the type of measurement – L: length, H: height, W: width, I: inclination (angle) – the second and third letters correspond to the part of the vertebra measured – c: centrum, na: neural arch, np: neural process (neural spine), m: metapophysis, tp: transverse process, ch: chevron. Figure adapted from Gillet et al. (2022)[28].

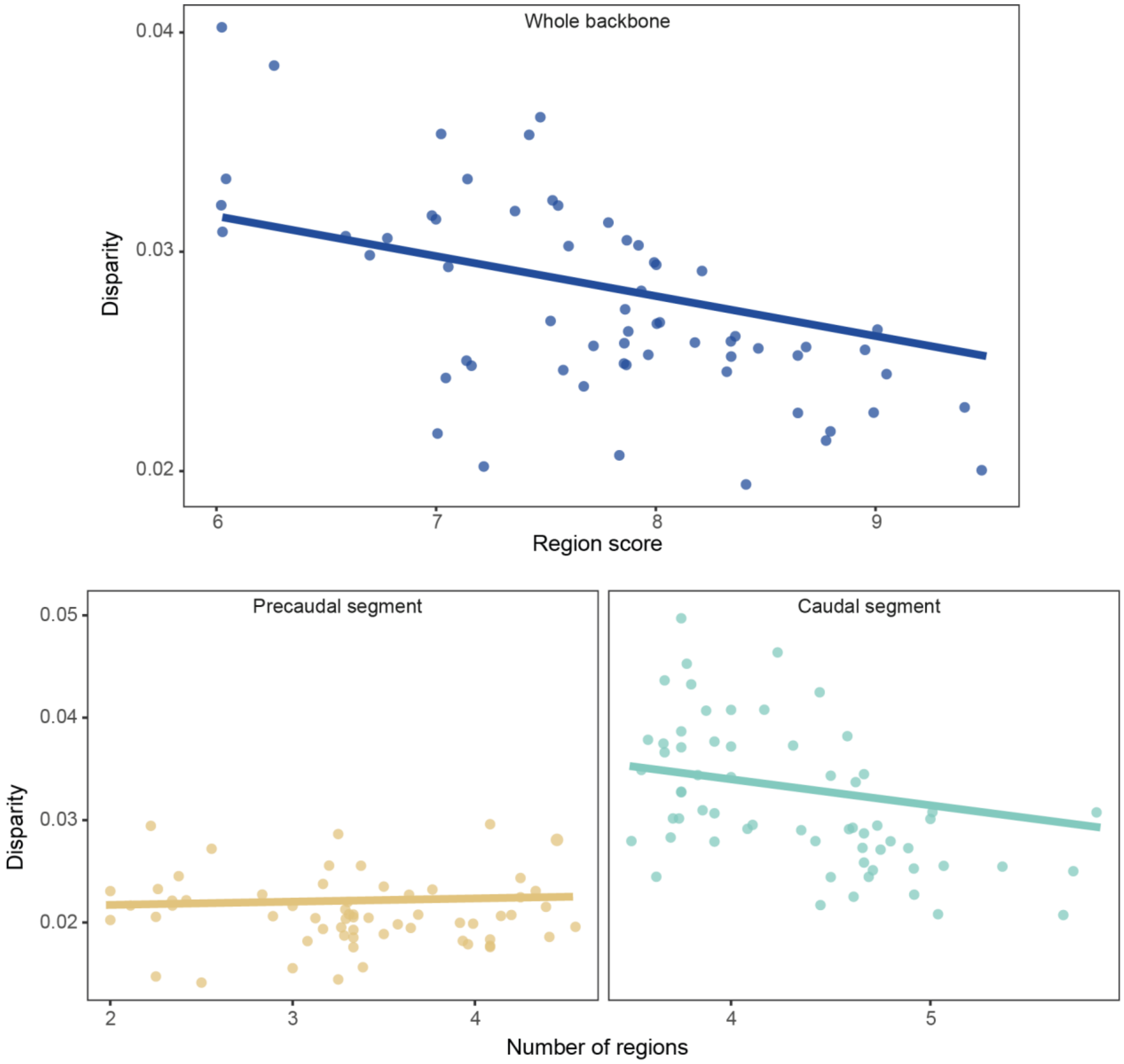

Supplementary Fig. 3. Relationship between regionalization and disparity. Linear regressions between the region score (whole backbone) or number of regions (precaudal and caudal segments) and disparity values. Disparity significantly decreases with increasing regionalization level in the entire backbone (*P* < 0.001) and in the caudal segment (*P* = 0.004) but not in the precaudal segment (*P* = 0.476). Source data are provided as a Source Data file.

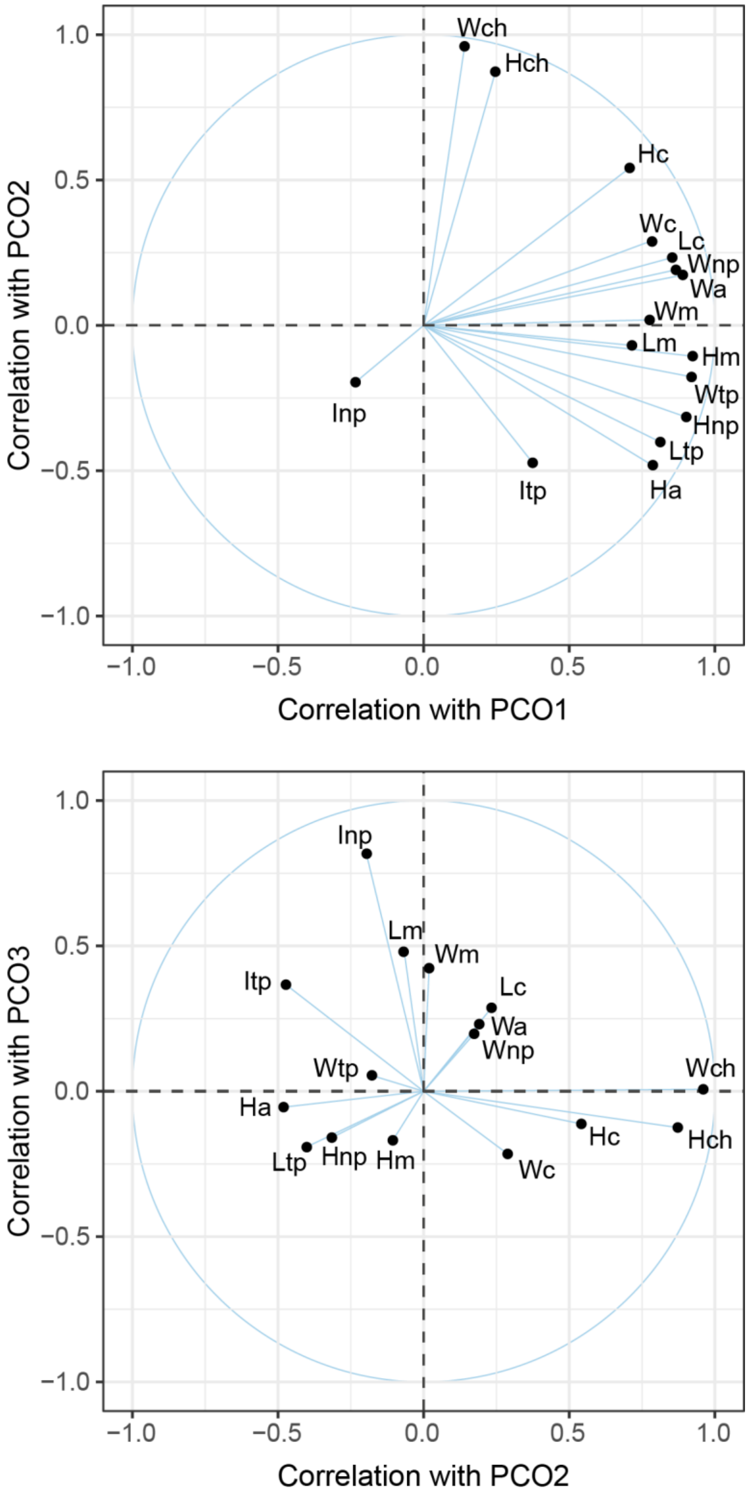

Supplementary Fig. 4. Variables contributions to PC axes. Correlations between vertebral shape measurements and PC axes 1-2 (top) and 2-3 (bottom) of the common morphospace of all specimens presented in Fig. 2a,b. Source data are provided as a Source Data file.

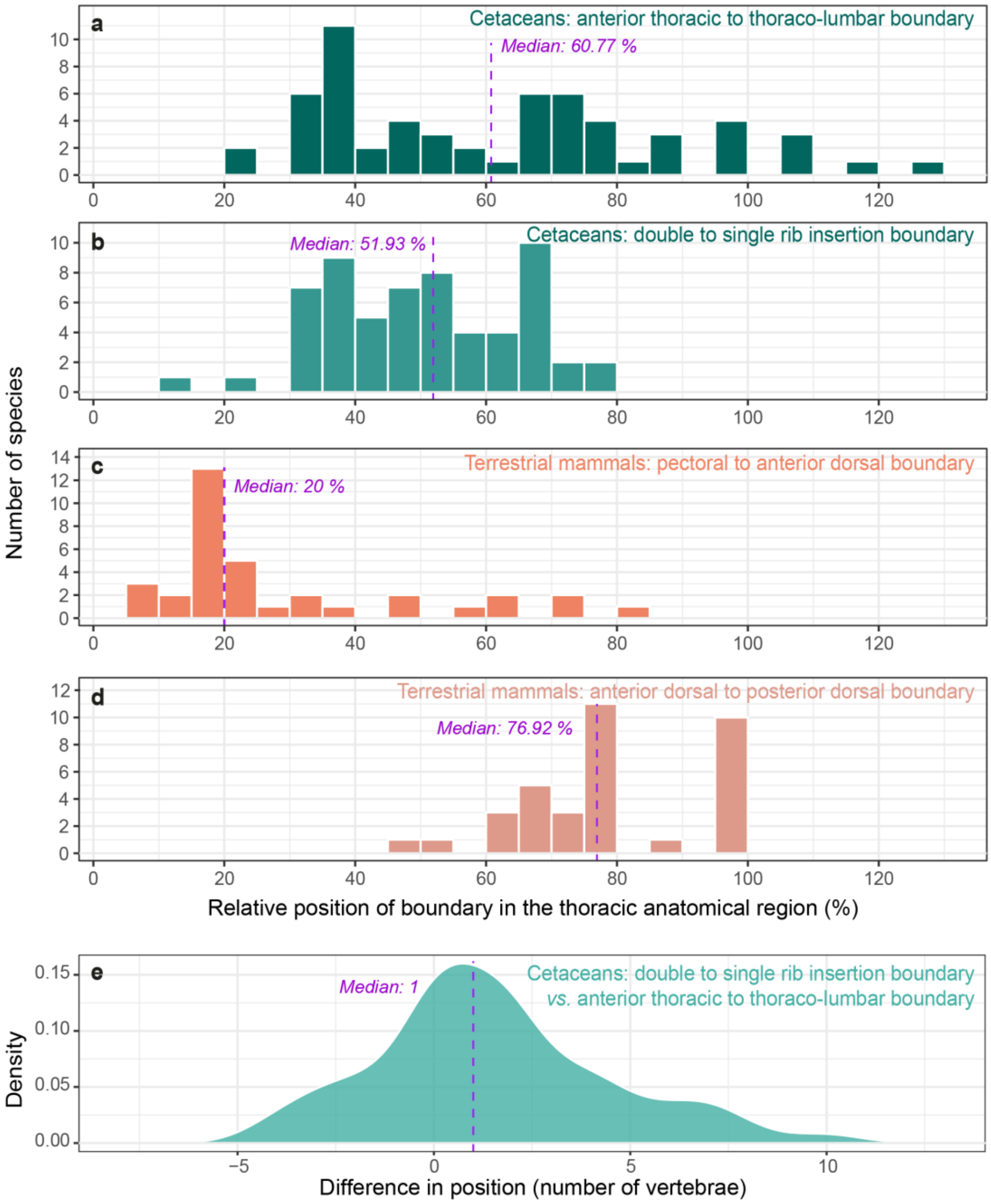

Supplementary Fig. 5. Relative position of anterior thoracic/thoraco-lumbar module boundary. a – d. Histograms of the positions of cetacean anterior thoracic to thoraco-lumbar module boundary (**a**), cetacean transition from double to single rib insertion (**b**), terrestrial mammals' pectoral to anterior dorsal boundary (**c**) and anterior dorsal to posterior dorsal boundary (**d**). Positions are expressed in percent of the total number of vertebrae in the traditional (rib-bearing) thoracic region. Terrestrial mammal data are from Jones et al. (2018)[9]. **e.** Distribution of differences (expressed in number of vertebrae) between the position of the transition from double to single headed ribs and the position of the cetacean anterior thoracic to thoraco-lumbar module boundary. In a majority of cetaceans, the boundary between the anterior thoracic and thoraco-lumbar modules falls close to the transition from double to single headed ribs. Positive values correspond to the module boundary being more posterior to the transition in rib insertion. The pectoral to anterior dorsal transition of terrestrial mammals tends to fall more anteriorly than the anterior thoracic to thoraco-lumbar transition of cetaceans, suggesting that the anterior thoracic module of cetaceans is not homologous to the pectoral region of terrestrial mammals. Source data are provided as a Source Data file.

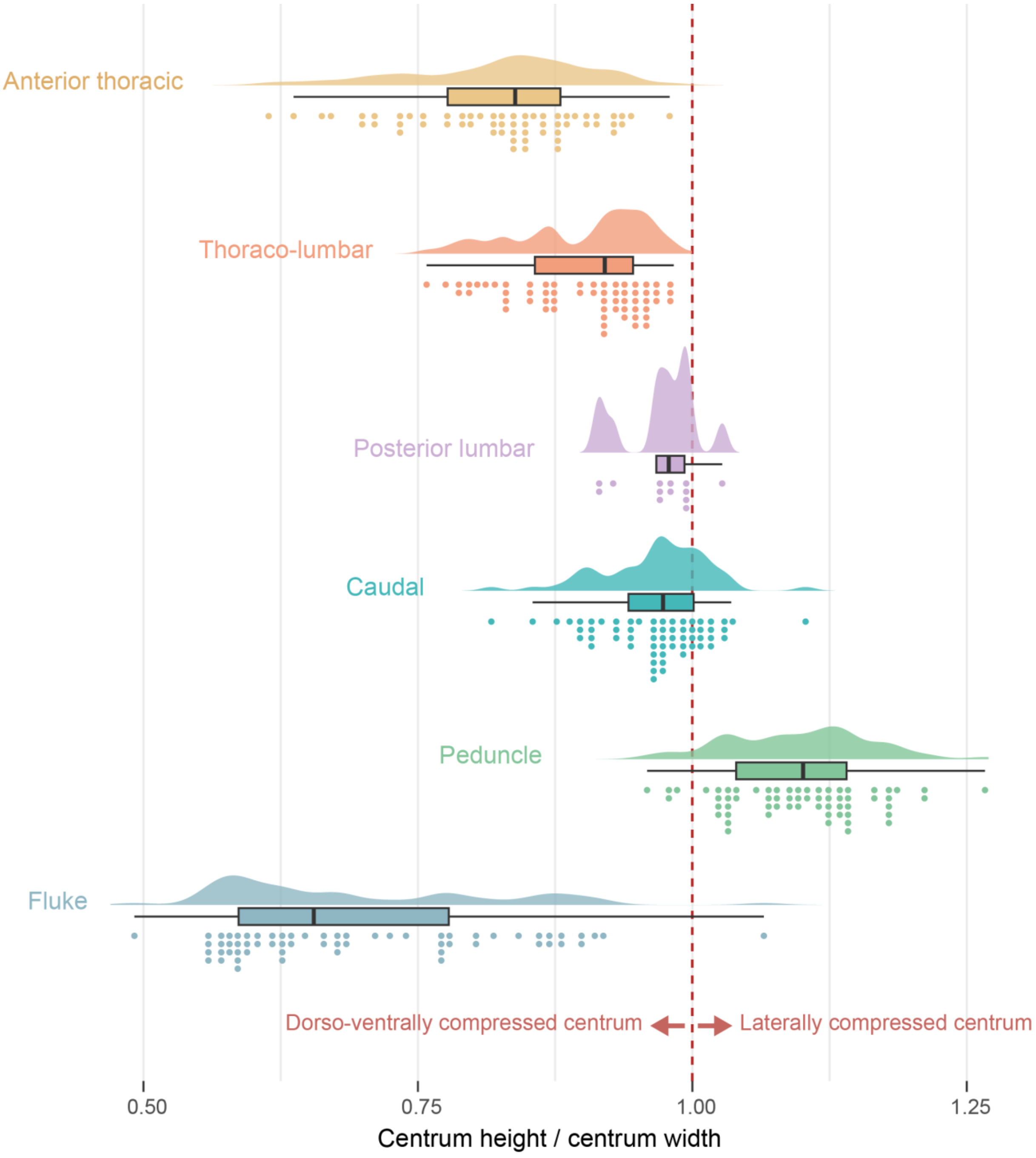

Supplementary Fig. 6. Average shape of vertebral centrum in modules. Distribution of the ratio between vertebral centrum height and width in each module. Ratio values equal to one correspond to a vertebral centrum with a circular cross section while values lower and greater than one correspond to dorso-ventrally and laterally compressed centrum, respectively. For each module, the density distribution (top), boxplot (middle), and distribution of data points (bottom) are presented. Each point corresponds to the average ratio of all vertebrae in the module of a given species. In boxplots, the median is represented by the center line, the first and third quartiles by the box limits, and the 1.5x interquartile range by whiskers. Source data are provided as a Source Data file.

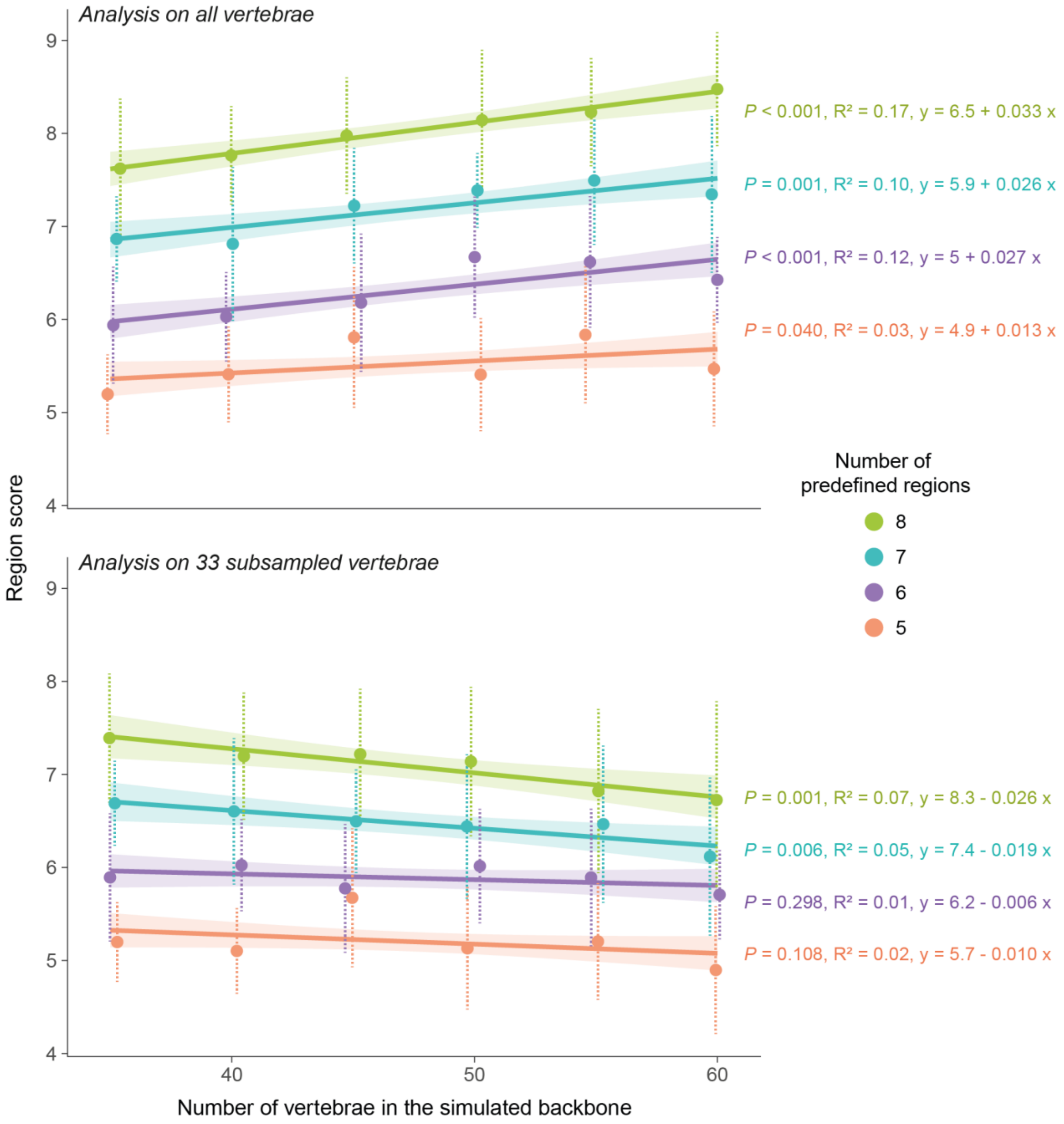

Supplementary Fig. 7. Results of sensitivity analysis on the effect of number of vertebrae included in the analyses. Regression between the region score recovered by the regionalization analyses on simulated backbones with a given number of vertebrae (ranging from 35 to 60) and number of predefined regions (ranging from 5 to 8). Top graph shows results from analyses run on all vertebrae, bottom graph shows results for analyses run on 33 subsampled vertebrae along the simulated backbone. Points correspond to the average region score of the 25 simulated backbones for a given number of vertebrae and predefined regions, dotted lines correspond to their standard deviation. Source data and exact regression coefficients are provided as a Source Data file.

Supplementary Table 1. Results of phylogenetically-corrected linear regressions between vertebral count, regionalization, and disparity. For each regression, lambda value was estimated with the *corPagel* function. Analyses were run on all species (n = 62).

**Regression** (independent vs. dependant variable)	**p-value**	**R^2**	**slope**	**lambda**
**Regionalization vs. disparity**				
Whole backbone	1.56E-258	0.55	-0.0018	1.02
Precaudal segment	0.476	0.50	0.0003	0.95
Caudal segment	0.004	0.52	-0.0026	0.99
**Number of vertebrae vs. regionalization**				
Whole backbone	6.97E-07	0.57	0.036	0.42
Precaudal segment	0.010	0.11	0.038	0.20
Caudal segment	2.44E-06	0.42	0.046	0.08
**Number of vertebrae vs. disparity**				
Whole backbone	1.11E-16	0.79	-0.00020	1.02
Precaudal segment	0.001	0.58	-0.00009	0.91
Caudal segment	2.09E-11	0.72	-0.00314	0.96

Supplementary Table 2. Effect of habitat on region score and vertebral disparity. Results from phylogenetically-corrected two-sided ANOVAs**.** For each regression, lambda value was estimated with the *corPagel* function. Species were divided into four habitat categories: rivers and bays (n = 7), coasts (n = 11), mixed (n = 8), and offshore (n = 36).

**Effect of habitat on:**	**F-value**	**p-value**	**lambda**
Region score	4.248	0.009	0.62
Disparity	9.192	4.56E-05	1.02

Supplementary Table 3. Results of phylogenetically-corrected linear regressions between region scores and swimming speeds. N: number of species on which each regression was calculated. For each regression, lambda value was estimated with the *corPagel* function.

**Effect of region score on:**	**N**	**p-value**	**R^2**	**slope**	**lambda**
Sustained swimming speed	34	0.188	0.47	0.143	0.47
Burst swimming speed	26	0.009	0.66	0.734	0.63
