# Supplementary material for: Repatterning of mammalian backbone regionalization in cetaceans
Source: Nat Commun. 2024 Aug 31;15:7587. doi: 10.1038/s41467-024-51963-w (PMC11365943; doi:10.1038/s41467-024-51963-w)
Supplement: Supplementary file 3 — Description of Additional Supplementary Files [file 41467_2024_51963_MOESM3_ESM.pdf]

# Description of Supplementary Data Files for:

## Repatterning of mammalian backbone regionalization in cetacean

Amandine Gillet[1,2], Katrina E. Jones[1], Stephanie E. Pierce[2]

*[1] Department of Earth and Environmental Sciences, University of Manchester, Williamson Building, Oxford Road, Manchester M13 9PL, UK*

*[2] Museum of Comparative Zoology and Department of Organismic and Evolutionary Biology, Harvard University, 26 Oxford Street, Cambridge, MA 02138, USA*

**Supplementary Data 1:** List of specimens sampled this study and their collection number. *N*: number of specimens per species. Institutional abbreviations: NRM: Swedish Museum of Natural History; SMNS: Stuttgart State Museum of Natural History; SAM: Iziko Museums of South Africa; PEM: Port Elizabeth Museum; USNM: Smithsonian National Museum of Natural History; AMNH: American Museum of Natural History. Names of curators and/or collection managers in charge of collections (at the time of data sampling) are listed in the second sheet of the file.

**Supplementary Data 2:** Raw vertebral measurements (in cm) of specimens sampled in this study. Each sheet contains measurements of a specimen with sheet names corresponding to abbreviated species names and specimen collection numbers. Abbreviations of linear and angular measurements as described in Supplementary Fig. 2. *Vert*: position of the vertebra in the backbone; *Vert_region*: position of the vertebra in its traditional anatomical region.

**Supplementary Data 3:** Ecological data and vertebral parameters per species. *Nbr vert.*: maximal number of vertebrae observed for the species across specimens in this study (supp. Table 1). *Ribs*: relative position (% of thoracic length) of transition from double to single headed ribs. Region score obtained from segmented linear regression analyses. *Disparity*: average distance between successive vertebrae along the backbone based on PCO scores. Detailed references for swimming speeds are presented in the second sheet.
