# Supplementary material for: Repatterning of mammalian backbone regionalization in cetaceans
Source: Nat Commun. 2024 Aug 31;15:7587. doi: 10.1038/s41467-024-51963-w (PMC11365943; doi:10.1038/s41467-024-51963-w)
Supplement: Supplementary file 7 — Reporting Summary [file 41467_2024_51963_MOESM7_ESM.pdf]

nature portfolio

Corresponding author(s): Amandine Gillet
Katrina E. Jones
Stephanie E. Pierce

Last updated by author(s): Aug 1, 2024

Reporting Summary

Nature Portfolio wishes to improve the reproducibility of the work that we publish. This form provides structure for consistency and transparency in reporting. For further information on Nature Portfolio policies, see our Editorial Policies and the Editorial Policy Checklist.

Statistics

For all statistical analyses, confirm that the following items are present in the figure legend, table legend, main text, or Methods section.

n/a	Confirmed	
☐	☒	The exact sample size (*n*) for each experimental group/condition, given as a discrete number and unit of measurement
☐	☒	A statement on whether measurements were taken from distinct samples or whether the same sample was measured repeatedly
☐	☒	The statistical test(s) used AND whether they are one- or two-sided<br>*Only common tests should be described solely by name; describe more complex techniques in the Methods section.*
☐	☒	A description of all covariates tested
☒	☐	A description of any assumptions or corrections, such as tests of normality and adjustment for multiple comparisons
☐	☒	A full description of the statistical parameters including central tendency (e.g. means) or other basic estimates (e.g. regression coefficient) AND variation (e.g. standard deviation) or associated estimates of uncertainty (e.g. confidence intervals)
☐	☒	For null hypothesis testing, the test statistic (e.g. F, t, r) with confidence intervals, effect sizes, degrees of freedom and P value noted<br>*Give P values as exact values whenever suitable.*
☒	☐	For Bayesian analysis, information on the choice of priors and Markov chain Monte Carlo settings
☐	☒	For hierarchical and complex designs, identification of the appropriate level for tests and full reporting of outcomes
☒	☐	Estimates of effect sizes (e.g. Cohen's *d*, Pearson's *r*), indicating how they were calculated

Our web collection on statistics for biologists contains articles on many of the points above.

Software and code

Policy information about availability of computer code

Data collection	No software was used for data collection
Data analysis	Data analysis was conducted in R (v.4.0.5) using the following packages: MorphoRegions (v.0.1.0), Spectrum (v.1.1), phytools (v.0.7-70), geiger (v.2.0.7), ape (v.5.4-1), nlme (v.3.1-152), rr2 (v.1.1.0).

For manuscripts utilizing custom algorithms or software that are central to the research but not yet described in published literature, software must be made available to editors and reviewers. We strongly encourage code deposition in a community repository (e.g. GitHub). See the Nature Portfolio guidelines for submitting code & software for further information.

Data

Policy information about availability of data

All manuscripts must include a data availability statement. This statement should provide the following information, where applicable:
- Accession codes, unique identifiers, or web links for publicly available datasets
- A description of any restrictions on data availability
- For clinical datasets or third party data, please ensure that the statement adheres to our policy

The list of specimens used in the study is available in Supplementary Data 1. Raw vertebral measurements generated in this study are available in Supplementary Data 2. Ecological categories, vertebral count, body size, and swimming speed gathered for each species are available in Supplementary Data 3. Data presented in Figures 2 to 5 and Supplementary Figures 3 to 7 are provided in a Source Data file.

Research involving human participants, their data, or biological material

Policy information about studies with human participants or human data. See also policy information about sex, gender (identity/presentation), and sexual orientation and race, ethnicity and racism.

Reporting on sex and gender	No human participants, data, or samples were involved in this study
Reporting on race, ethnicity, or other socially relevant groupings	No human participants, data, or samples were involved in this study
Population characteristics	No human participants, data, or samples were involved in this study
Recruitment	No human participants, data, or samples were involved in this study
Ethics oversight	No human participants, data, or samples were involved in this study

Note that full information on the approval of the study protocol must also be provided in the manuscript.

Field-specific reporting

Please select the one below that is the best fit for your research. If you are not sure, read the appropriate sections before making your selection.

☐ Life sciences ☐ Behavioural & social sciences ☒ Ecological, evolutionary & environmental sciences

For a reference copy of the document with all sections, see nature.com/documents/nr-reporting-summary-flat.pdf

Ecological, evolutionary & environmental sciences study design

All studies must disclose on these points even when the disclosure is negative.

Study description	Study investigating the regionalization patterns of the cetacean backbone and its relation with evolutionary history, habitat, and swimming performances.
Research sample	Sample includes vertebral measurements of 139 specimens from 62 extant cetacean species. Specimens were sampled to encompass the largest diversity of cetaceans possible based on the availability of complete vertebral columns in museums.
Sampling strategy	The sample represents more than 65% of extant cetacean species, with at least one species sampled in each major clade/family. Because sof the large size and/or scarcity of some species, between 1 and 10 specimens were sampled for each species depending on availability.
Data collection	Vertebral measurements were collected directly on osteological specimens in museums using digital calipers, rulers, and protractors. Data on habitat and swimming speeds were collected from the litterature. All data were collected by AG.
Timing and spatial scale	Morphological data were collected between 2016 and 2018.
Data exclusions	No data were excluded from the analyses
Reproducibility	When possible, data were collected on multiple specimens from the same species and analyses were run on all specimens, accounting for intraspecific variability.<br>All data and code to reproduce analyses are available in Methods, Supplementary Information, Supplementary Data, and on Github.
Randomization	Specimens were grouped by species. To test the effect of habitat, species were divided in four habitat categories (rivers, coastal, offshore, mixed between coastal and offshore) based on data available in the litterature.
Blinding	Blinding was not possible as it was necessary to identify species sampled to ensure a large coverage of cetacean diversity

Did the study involve field work? ☐ Yes ☒ No

Reporting for specific materials, systems and methods

We require information from authors about some types of materials, experimental systems and methods used in many studies. Here, indicate whether each material, system or method listed is relevant to your study. If you are not sure if a list item applies to your research, read the appropriate section before selecting a response.

Materials & experimental systems

n/a	Involved in the study
☒	☐ Antibodies
☒	☐ Eukaryotic cell lines
☒	☐ Palaeontology and archaeology
☐	☒ Animals and other organisms
☒	☐ Clinical data
☒	☐ Dual use research of concern
☒	☐ Plants

Methods

n/a	Involved in the study
☒	☐ ChIP-seq
☒	☐ Flow cytometry
☒	☐ MRI-based neuroimaging

Animals and other research organisms

Policy information about studies involving animals; ARRIVE guidelines recommended for reporting animal research, and Sex and Gender in Research

Laboratory animals	The study did not involve laboratory animals
Wild animals	The study did not involve wild animals
Reporting on sex	Information on sex of osteological specimens is not consistently available. Where possible, at least one specimen of each sex was sampled for each species. Due to the scarcity of information on sex for cetacean specimens with fully complete backbone, no test was performed to assess the effect of sex.
Field-collected samples	The study did not involve field-collected samples, only samples from osteological collections
Ethics oversight	No ethical approval was required, all specimens were already accessioned in collections prior to study

Note that full information on the approval of the study protocol must also be provided in the manuscript.

Plants

Seed stocks	*Report on the source of all seed stocks or other plant material used. If applicable, state the seed stock centre and catalogue number. If plant specimens were collected from the field, describe the collection location, date and sampling procedures.*
Novel plant genotypes	*Describe the methods by which all novel plant genotypes were produced. This includes those generated by transgenic approaches, gene editing, chemical/radiation-based mutagenesis and hybridization. For transgenic lines, describe the transformation method, the number of independent lines analyzed and the generation upon which experiments were performed. For gene-edited lines, describe the editor used, the endogenous sequence targeted for editing, the targeting guide RNA sequence (if applicable) and how the editor was applied.*
Authentication	*Describe any authentication procedures for each seed stock used or novel genotype generated. Describe any experiments used to assess the effect of a mutation and, where applicable, how potential secondary effects (e.g. second site T-DNA insertions, mosiacism, off-target gene editing) were examined.*
